# Concentrations of Water-Soluble Forms of Choline in Human Milk from Lactating Women in Canada and Cambodia

**DOI:** 10.3390/nu10030381

**Published:** 2018-03-20

**Authors:** Alejandra M. Wiedeman, Kyly C. Whitfield, Kaitlin M. March, Nancy N. Chen, Hou Kroeun, Ly Sokhoing, Prak Sophonneary, Roger A. Dyer, Zhaoming Xu, David D. Kitts, Timothy J. Green, Sheila M. Innis, Susan I. Barr

**Affiliations:** 1 Food, Nutrition, and Health Program, The University of British Columbia, Vancouver, BC V6T 1Z4, Canada; awiedeman@bcchr.ca (A.M.W.); kyly.whitfield@msvu.ca (K.C.W.); kaitlin.march@cw.bc.ca (K.M.M.); nnchen30@gmail.com (N.N.C.); zhaoming.xu@ubc.ca (Z.X.); david.kitts@ubc.ca (D.D.K.); tim.green@sahmri.com (T.J.G.); 2 BC Children’s Hospital Research Institute, Vancouver, BC V5Z 4H4, Canada; radyer@mail.ubc.ca (R.A.D.); sinnis@mail.ubc.ca (S.M.I.); 3 Department of Applied Human Nutrition, Mount Saint Vincent University, Halifax, NS B3M 2J6, Canada; 4 Helen Keller International—Cambodia Country Office, Phnom Penh 12301, Cambodia; hkroeun@hki.org (H.K.); lsokhoing@hki.org (L.S.); 5 National Nutrition Programme, Maternal and Child Health Centre, Ministry of Health, Phnom Penh 12202, Cambodia; sophonprak@gmail.com; 6 South Australia Health and Medical Research Institute, Adelaide, SA 5000, Australia

**Keywords:** choline, phosphocholine, glycerophosphocholine, lactation, human milk, infants, adequate intake, dietary recommendations, Canada, Cambodia

## Abstract

Choline has critical roles during periods of rapid growth and development, such as infancy. In human milk, choline is mostly present in water-soluble forms (free choline, phosphocholine, and glycerophosphocholine). It is thought that milk choline concentration is influenced by maternal choline intake, and the richest food sources for choline are of animal origin. Scarce information exists on milk choline from countries differing in animal-source food availability. In this secondary analysis of samples from previous trials, the concentrations of the water-soluble forms of choline were quantified by liquid chromatography-tandem mass spectrometry in mature milk samples collected from lactating women in Canada (*n* = 301) and in Cambodia (*n* = 67). None of the water-soluble forms of choline concentrations in milk differed between Canada and Cambodia. For all milk samples (*n* = 368), free choline, phosphocholine, glycerophosphocholine, and the sum of water-soluble forms of choline concentrations in milk were (mean (95%CI)) 151 (141, 160, 540 (519, 562), 411 (396, 427), and 1102 (1072, 1133) µmol/L, respectively. Theoretically, only 19% of infants would meet the current Adequate Intake (AI) for choline. Our findings suggest that the concentrations in milk of water-soluble forms of choline are similar in Canada and Cambodia, and that the concentration used to set the infant AI might be inaccurate.

## 1. Introduction

Choline is an essential nutrient with important roles during periods of rapid growth and development, such as early infancy [1,2,3]. Choline has a wide range of functions, including serving as a precursor for the synthesis of acetylcholine, phospholipids critical for cell membranes, surfactants important in lung maturity, bile formation, and betaine, which is an osmolyte and a methyl group donor [4,5]. In humans, large amounts of choline are present in milk, mainly as the water-soluble forms free choline, phosphocholine, and glycerophosphocholine, contributing an average of 84% of the total choline, whereas the lipid-soluble (phosphatidylcholine and sphingomyelin) forms account for the remaining 16% [6,7,8,9,10]. It has been reported that milk choline concentrations are positively associated with circulating choline concentrations in infants [7], and elevated circulating choline, as free choline, might enhance free choline uptake across the blood-brain barrier [11,12]. The authors of a recent observational study suggest that higher milk free choline concentration (mean 158 µmol/L) is associated with better infant recognition memory (latency at central leads, β = 1.90, R^2^ = 0.30, *p* < 0.01) adjusted by other milk nutrients at an age of six months [13].

The World Health Organization recommends exclusive breastfeeding for the first six months to promote optimal growth of the infant [14]. Generally, the average concentration of nutrients in mature milk from healthy, well-nourished mothers, in conjunction with the average volume consumed by fully breast-fed infants, is used to set dietary recommendations for infancy and to determine the additional amount of the nutrient over the recommendation for non-lactating women for lactation [1]. For early infancy (0–6 months), the Adequate Intake (AI) for choline was set at 125 mg/day (equivalent to 1500 µmol/L and an assumed volume of 780 mL) [1]. Milk choline concentrations are thought to reflect habitual maternal dietary choline intake [15,16], with foods of animal origin being the richest source of choline [17].

Limited data are available on choline concentration in mature human milk, including reports from the United States [6,8,10,18], Turkey [7,9], Canada [19], Japan [20], Korea [21], Sweden [22], and Ecuador [15]. Nevertheless, sample sizes in these studies are small (*n* = 1–75), and include milk samples collected after a full-term, pre-term, or mixed birth term pregnancy. Moreover, the available data on milk choline are generally from high-income countries where maternal animal-source food availability [23] and consumption is greater than in middle- and low-income countries [24,25].

In this study, we used secondary data from recent randomized controlled trials (none of which provided choline), in which mature milk samples were collected from lactating women in Canada and Cambodia, two countries differing in dietary food source availability [23]. Our primary aim was to assess the concentration of the water-soluble forms of choline. In addition, we also explored the associations between maternal dietary choline intake and milk concentrations of water-soluble forms of choline in a subset of the Canadian participants. We found that the concentrations of milk water-soluble forms of choline did not significantly differ between Canada and Cambodia, despite lower likely choline intakes in Cambodia, and that maternal total dietary choline intake was not significantly associated with the concentration of water-soluble forms of choline in milk in a subset of the Canadian participants.

## 2. Materials and Methods

### 2.1. Study Participants and Sample Collection

The present study was a secondary cross-sectional analysis of previous randomized controlled trials conducted in Canada and Cambodia [26,27,28]. In all the trials, convenience samples of mature milk were collected from apparently healthy lactating women (18–45 year) who had low-risk pregnancies, uncomplicated deliveries, and gave birth to healthy, full-term infants. All subjects gave their informed consent for inclusion before they participated in the studies. The studies were conducted in accordance with the Declaration of Helsinki. Demographic characteristics including age, ethnicity, education, and household income were collected from the participants.

#### 2.1.1. Canadian Samples

In Canada, participants were enrolled in two supplementation trials (neither of which contained choline) and have been previously described [26,27]. In the first trial, women had consumed a daily supplement containing docosahexaenoic acid or placebo from 16 weeks gestation through to the end of pregnancy [26]. In the second trial, women had consumed a daily prenatal multivitamin and mineral supplement and were randomly assigned to one of three vitamin D supplement groups between 13–24 weeks gestation through to eight weeks postpartum [27]. The University of British Columbia Children’s and Women’s Research Ethics Board approved these trials (H08-70242 and H09-01261).

Canadian participants provided a single mature milk sample at eight weeks postpartum. In the first trial, hindmilk samples were collected at the participant’s house into pre-labeled tubes and stored in a home freezer for up to three days before being stored at −80 °C [26]. For the present study, a total of 147 milk samples from the first trial were available for analysis. In the second Canadian trial, a full breast expression from the breast that had not been most recently emptied was collected during a clinical visit and stored at −80 °C [27]. For the present study, a total of 154 milk samples from the second trial were available for analysis.

#### 2.1.2. Cambodian Samples

In Cambodia, women had been consuming either a thiamine-fortified or placebo fish sauce, containing insignificant choline content [17], ad libitum for six months before milk collection [28]. The Cambodian National Ethics Committee for Health Research (245 NECHR) approved this trial.

Participants provided a single mature milk sample between three to 28 weeks postpartum as a full breast expression from the breast that had not been most recently emptied in their villages in Prey Veng. The milk samples were transported on ice to the National Institute of Public Health in Phnom Penh (<5 h) for storage at −80 °C until being shipped on dry ice to the University of British Columbia for analysis. For the present study, a total of 67 milk samples from Cambodia were available for analysis.

### 2.2. Biochemical Analysis

For all milk samples (*n* = 368), the concentrations of the water-soluble forms of choline (free choline, phosphocholine, and glycerophosphocholine) were quantified in 20 μL aliquots by stable isotope dilution liquid chromatography-tandem mass spectrometry (LC-MS/MS), according to the method described in detail elsewhere [19]. The inter-assay and intra-assay coefficients of variation were: 4.8% and 1.4% for free choline, 5.4% and 0.2% for phosphocholine, and 3.6% and 2.1% for glycerophosphocholine, respectively.

### 2.3. Dietary Choline Intake Assessment

For all infants (*n* = 368), dietary choline intake was estimated using milk total choline concentration and daily reference intake volume, based on the methods used to set the AI for early infancy [1]. First, total choline in milk was calculated by multiplying the concentration of the sum of the water-soluble forms of choline of each participant by a conversion factor (1.19), based on the assumption that on average the water-soluble forms of choline make up 84% of total choline in human milk [6,7,8,9,13]. Then, dietary choline intake from milk was estimated using 780 mL/day as the reference volume of milk consumption of breastfed infants during their first six months [1].

In Canada, dietary intake during lactation was available for 143 of the participants enrolled in the first trial. Intake was estimated using a semi-quantitative food frequency questionnaire (FFQ), covering the intake of the previous month, administered at 16 and 36 weeks of gestation. Dietary intakes of choline were estimated using a nutrient analysis software (ESHA Food Processor SQL, version 10.14.41; Salem, OR) and the United States Department of Agriculture (USDA) database on the Choline Content of Common Foods (Version 2) [17]. A validation analysis indicated that compared to the mean of three 24 h recalls, the FFQ was a valid instrument to assess dietary choline intake: the energy-adjusted de-attenuated correlation coefficient was 0.70, only 7.5% were grossly misclassified (e.g., opposite tertiles), and weighted Cohen’s kappa was 0.32 [29].

### 2.4. Statistical Analysis

All statistical analyses were performed using SPSS 22.0 (SPSS Inc., Chicago, IL, USA) and statistical significance was set at *p* < 0.05 for two-sided testing. Data normality was determined using the Kolmogorov-Smirnov test, and skewed distributed data was log-transformed before further analyses. The concentrations of water-soluble forms of choline in milk were compared within each trial, by randomization groups, using independent samples one-way analysis of variance (ANOVA) or Student’s *t*-test, as appropriate. In Canada, the concentrations of water-soluble forms of choline in milk were compared between the two trials, by milk fraction collected (i.e., hindmilk versus a full breast expression), using independent samples Student’s *t*-test. In Cambodia, the relationship between the concentration of water-soluble forms of choline in milk and weeks postpartum was assessed by Pearson’s correlation coefficient.

Demographic characteristics including age, ethnicity, education, and household income were compared between the two groups, Canada and Cambodia, using independent samples Student’s *t*-test for continuous variable and Fisher’s exact test for categorical variables. Water-soluble forms of choline concentrations in milk were compared between Canadian and Cambodian participants using independent samples Student’s *t*-test. The estimated total choline intake was compared with the corresponding AI by one sample Student’s *t*-test. The relationships between maternal dietary choline intake during pregnancy and the concentrations of water-soluble forms of choline in milk samples at eight weeks postpartum were explored using Pearson’s correlation coefficient, and Bonferroni’s correction was used to adjust for multiple comparisons.

## 3. Results

A total of 301 milk samples from Canadian and 67 milk samples from Cambodian lactating women were included in this analysis. In Canada, the concentrations of individual and total water-soluble forms of choline in milk compared within each trial (randomization groups) and between the two trials (milk fraction collected) did not differ (*p >* 0.05 for all, Table S1), and therefore the participants from both Canadian trials are presented as one group. Among the Cambodian sample, the concentrations of individual and total water-soluble forms of choline in milk compared by randomization groups did not differ, and there was no need to control for weeks postpartum, as no statistically significant correlations were found with the concentrations of individual and total water-soluble forms of choline in milk (*p* > 0.05 for all, Table S2). Participant characteristics are shown in Table 1. Compared to Cambodian women, Canadian women differed in age, education, ethnicity, and household income (*p* < 0.001).

The concentrations of the individual and the sum of the water-soluble forms of choline in milk are presented in Table 2, and did not differ significantly when comparing milk samples between Canada and Cambodia (*p >* 0.05, for all). Although Canadian women were older than Cambodian women, maternal age was not correlated with milk choline concentrations (r = 0.014 to 0.065, *p* > 0.05, for all). Among all samples (*n* = 368), phosphocholine and glycerophosphocholine were the predominant compounds, contributing 49% and 37% to the sum of the water-soluble forms of choline in milk, respectively.

For infants, the estimated total choline intakes in Canada, Cambodia, and in both countries combined are presented in Table 3, and were significantly below the AI for choline for early infancy (125 mg/day; *p* < 0.001, for all). Based on these data, only 19% of infants in Canada and Cambodia would have met the current choline AI recommendation, compared to an expected value of ~50% [1]. For maternal intake, since the estimated choline intakes between gestational time points (16 and 36 weeks of gestation) did not differ (*p* = 0.927, Table S3) and were significantly correlated (r = 0.590, *p* < 0.001), only the second time point was used, as it was the closest to the milk collection. The estimated maternal total choline intake was significantly below the AI for choline for both pregnancy (450 mg/day) and lactation (550 mg/day) (*p* < 0.001 for both).

The correlation coefficients between the estimated choline intake and the concentrations of the water-soluble forms of choline in milk are presented in Table 4. The sum of the water-soluble forms of choline in milk was positively, but weakly, correlated with dietary total choline intake (r = 0.166, *p* = 0.048), dietary free choline intakes (r = 0.223, *p* = 0.007), and dietary water-soluble forms of choline intake (r = 0.182, *p* = 0.029). After adjusting for multiple comparisons, the correlations were no longer significant (Bonferroni’s correction *p* > 0.006, for all).

## 4. Discussion

An interesting finding from our study was that concentrations of the water-soluble forms of choline in mature milk did not differ between lactating women in Canada and Cambodia (1106 and 1095 µmol/L, respectively), regardless of differences in animal food availability [23] and different likely dietary choline intakes, as described for other low- and middle-income countries [24,25]. An earlier study, in 1982, reported that free choline concentration in milk was lower in lactating women in Ecuador (40% < 100 µmol/L) compared to the United States (55% > 300 µmol/L) [15]. However, free choline contributes an average of only 10% of the total choline in human milk [6,7,8,9,10], and it is unknown whether this difference would be physiologically relevant. Although dietary information was not collected, the authors hypothesized that the difference was a result of the low dietary choline intake in Ecuador [15].

Two recent choline supplementation studies reported that lactating women consuming choline supplemented diets (means of 930 or 1088 mg/day) with close to two-times the corresponding AI (550 mg/day) had significantly higher milk total choline concentrations (20%) compared to control groups (mean intakes of 364 or 480 mg/day) [8,10]. In our study, the mean dietary choline intake in Canada (408 mg/day) was closer to the amount of choline consumed in the control than the supplemented groups of previous studies [8,10]. Thus, the difference in the dietary choline intake between Canada and Cambodia may not have reached the range in which a difference in milk choline concentration would be observed. An alternative explanation for similar milk choline concentrations between Canadian and Cambodian women in our study may be that the mammary gland can obtain choline from both maternal circulation [30] and the endogenous synthesis of phosphatidylcholine through the phosphatidylethanolamine *N*-methyl transferase (PEMT) pathway [31]. Although it has been described that PEMT is mostly active in liver and kidney [32,33], its activity has also been identified in the epithelial cells of the mammary gland [34]. Consequently, this may contribute to ensuring that sufficient choline is excreted in milk to meet the choline demand for the rapidly growing and developing infant.

The estimated mean concentration of water-soluble forms of choline reported in our study was 1102 µmol/L (*n* = 368), which is in agreement with previously reported values in mature milk samples. In 1996, the first study including all individual forms of choline in mature milk from the United States reported a concentration of water-soluble forms of choline of 1048 µmol/L (*n* = 16) [6]. Two later studies from the same country have reported mean concentrations of water-soluble forms of choline of 987 and 1024 µmol/L (*n* = 28 and *n* = 48) [8,10], where choline quantification was conducted by LC-MS/MS as in our analysis. In Canada, our group recently reported on the concentration of water-soluble forms of choline in donor’s milk with a slightly higher mean concentration of water-soluble forms of choline of 1275 µmol/L (*n* = 30) [19]. However, it is unknown whether the pasteurization process, which is routinely used in milk banks, has an impact on the total or individual forms of choline concentration in milk. In Turkey, slightly higher concentrations of water-soluble forms of choline in mature milk (1189 and 1402 µmol/L) were reported in two studies (*n* = 12, *n* = 54), where choline was analyzed by less sensitive enzymatic methods [7,9].

Based on our results, the estimated total choline intake of the infants was 106 mg/day (*n* = 368), which is significantly below the corresponding AI for choline (125 mg/day) [1]. However, it is important to note that the AI for choline for infants of 0–6 months reflected a milk total choline concentration of 1500 µmol/L, which in fact was 20% higher than the mean concentration of total choline in human milk published at that time (1254 µmol/L) [6], and no rationale was provided as to why a higher concentration was used to set the AI during infancy instead of the observed average as per definition [35]. Accordingly, results categorized using the current AI for choline for infants of 0–6 months as a cut-off must be interpreted with caution, as the current recommendation may not accurately reflect the mean total choline concentration in human milk.

The milk choline concentrations reported in the present study are also relevant from a clinical perspective, as the nutrient content in human milk may be used as a guideline to develop human milk substitutes and enteral formulas for infants. Current guidelines for choline content in infant formula are 7–50 mg/100 kcal [36,37], which is equivalent to a wide range of choline intake ranging between 37–265 mg/day, based on milk consumption of 780 mL/day and energy content of 68 kcal/100 mL [36]. Most of the commercially available infant formulas add choline as free choline, which contributes 57% of total choline [17], compared to only 10% in human milk [6,7,8,9,10]. Accordingly, current infant formula composition does not mimic the profile of choline forms present in human milk; whether this is physiologically relevant is not known.

In this study, we found that the concentration of water-soluble forms of choline in milk was not significantly associated with maternal choline intake when estimated during pregnancy from a subset of the Canadian participants. In line with this finding, another study reported that choline intake was not associated with choline concentration in milk, except for a weak association with milk phosphatidylcholine (R^2^ = 0.11, *p* = 0.007) [8]. It also could be possible that maternal intake during pregnancy was not the same as when the milk samples were collected at eight weeks postpartum. However, previous studies have observed a minor and non-significant variation in the maternal diet when comparing intakes during pregnancy and lactation periods (2–4% change) [38,39]. This is in agreement with the finding that total choline intakes at 16 and 36 weeks of gestation did not differ (*p* = 0.927) and were positively associated (r = 0.590, *p* < 0.001) in our study.

A potential limitation of the present study was that milk samples were collected at different time points during lactation among the Cambodian participants (3–28 weeks postpartum). However, it has been described that total choline concentration in milk increases from colostrum at birth to two weeks postpartum, and then stays stable beyond six months [7,20,21,40], and we found no association between total water-soluble choline concentration and week postpartum of the milk collection (*p* = 0.915). Additionally, we compared different milk fractions, hindmilk versus a full breast expression, which, at least for the water-soluble forms of choline, did not differ in the Canadian trials (Table S1, *p* > 0.05) and is in agreement with an earlier study that compared different milk fractions [18]. Another limitation was that only the water-soluble forms of choline were quantified, without including the lipid-soluble forms. However, the water-soluble forms of choline contribute an average of 84% of the total choline concentration in human milk, which is relatively stable based on the published data even among the supplemented groups [6,7,8,9,10]. In addition, the actual infant daily volume intake of human milk is unknown, and the reference intake volume (780 mL/day) used to set the AI for different nutrients, including choline, for early infancy was selected [1], which was obtained from earlier studies assessing infant’s body weight before and after breastfeeding during the first six months of life [41,42,43,44]. Recently, a comparable value of the daily volume of human milk intake between two and five months has been published [45]. Moreover, no dietary information was collected in Cambodia and further studies need to be conducted to confirm the differences assumed in total choline intake.

## 5. Conclusions

The present study is the first to report water-soluble forms of choline concentrations in mature milk samples collected from lactating women in Cambodia and could be considered the largest study to date for choline assessment in human milk. In summary, we found that the concentrations of the water-soluble forms of choline in mature human milk samples did not differ between Canadian and Cambodian lactating women, despite lower likely choline intakes in Cambodia. The finding that the estimated dietary choline intake during early infancy was below the corresponding AI suggest that the current AI may not reflect the average choline concentration in human milk of healthy lactating women. Among a subset of the Canadian participants, maternal dietary choline was not significantly correlated to the milk water-soluble forms of choline.

## Figures and Tables

**Table 1:** Demographic characteristics of lactating women in Canada and Cambodia.

Demographic Characteristics	Canada (*n* = 301)	Cambodia (*n* = 67)	*p* Value ^2^
Age (year) ^1^	33.3 ± 4.1	26.1 ± 4.7	<0.001
Parity (*n*; %) 1 2 3 or more	161; 53% 112; 37% 28; 9%	34; 51% 24; 36% 9; 12%	0.720
Education (*n*; %) None Some primary Some secondary Some postsecondary	0; 0% 0; 0% 11; 4% 290; 96%	6; 9% 31; 46% 30; 44% 0; 0%	<0.001
Ethnicity (*n*; %) European Asian Khmer Other	234; 78% 40; 13% - 27; 9%	- - 67; 100% -	<0.001
Household income (*n*; %)			<0.001
<$20 000 CAD $20 000–50 000 CAD >$50 000 CAD	10; 3% 47; 16% 244; 81%	67; 100% - -	

^1^ Data are presented as mean ± SD; ^2^ Continuous data were analyzed by independent samples Student’s *t*-test, and categorical data analyzed by Fisher’s exact test.

**Table 2:** Concentration of water-soluble forms of choline in milk samples of lactating women in Canada and Cambodia ^1^.

Forms of Choline Concentration (µmol/L)	All Women (*n* = 368)	Canada (*n* = 301)	Cambodia (*n* = 67)	*p* Value ^2^
Free choline	151 (141, 160)	155 (144, 165)	143 (132, 154)	0.071
Phosphocholine	540 (519, 562)	535 (512, 559)	562 (513, 612)	0.336
Glycerophosphocholine	411 (396, 427)	416 (399, 434)	390 (356, 423)	0.178
Water-soluble choline ^3^	1102 (1072, 1133)	1106 (1071, 1140)	1095 (1018, 1150)	0.686

^1^ Data are presented as mean (95% CI), and concentrations were quantified using liquid chromatography-tandem mass spectrometry; ^2^ Group differences were analyzed by independent samples Student *t*-test after log-transformation; ^3^ Water-soluble choline corresponds to the sum of free choline, phosphocholine, and glycerophosphocholine.

**Table 3:** Estimated dietary total choline intake in Canada and Cambodia ^1^.

Dietary Intake	Infants ^2^			Maternal Canada (*n* = 143) ^3^
	All (*n* = 368)	Canada (*n* = 301)	Cambodia (*n* = 67)	
Total choline (mg/day)	106 (103, 109)	107 (103, 110)	105 (98, 111)	408 (390, 427)
AI (mg/day) Above AI ^4^ (*n*; %)	125 71; 19	125 58; 19	125 13; 19	450 46; 32

^1^ Dietary intake data are presented as mean (95% CI); ^2^ Total choline intakes were estimated assuming that water-soluble forms of choline contribute 84% to total choline and a reference milk intake of 780 mL/day; ^3^ Total choline intakes were estimated at 36 weeks of gestation using a food frequency questionnaire and the USDA database on choline content in common foods (version 2); ^4^ Since the AI reflects the average choline concentration of breastmilk from healthy lactating women, it is expected that ~50% of infants would have intakes above the AI. When mean intake equals the AI, it is assumed that the prevalence of inadequacy in the group is low; when mean intake is below the AI, no conclusions regarding adequacy can be drawn.

**Table 4:** Correlation between dietary choline intake during pregnancy and water-soluble forms of choline in milk from a subset of Canadian participants ^1^.

Dietary Intakes (*n* = 143)	Milk Choline Metabolites			
	Free Choline	Phospho-Choline	Glycerophospho-Choline	Water-Soluble Choline
Free choline	0.054	0.125	0.129	0.223 **
Phosphocholine	0.134	0.063	−0.007	0.128
Glycerophosphocholine	0.105	0.034	0.101	0.130
Water-soluble choline ^2^	0.102	0.094	0.093	0.182 *
Phosphatidylcholine	−0.016	0.145	−0.056	0.091
Sphingomyelin	−0.031	0.147	−0.109	0.036
Lipid-soluble choline ^3^	−0.016	0.150	−0.058	0.093
Total choline ^4^	0.043	0.150	0.017	0.166 *

^1^ Data are presented as Pearson’s correlation coefficients after log-transformation, none of the observed associations were significant after Bonferroni’s correction (0.05/8 = 0.006; *p* > 0.006, for all comparisons); ^2^ Water-soluble choline corresponds to the sum of free choline, phosphocholine, and glycerophosphocholine; ^3^ Lipid-soluble choline corresponds to the sum of phosphatidylcholine and sphingomyelin; ^4^ Total choline corresponds to the sum of all individual forms of choline; * *p* < 0.05, ** *p* < 0.01.

## Supplementary Materials

The following are available online at http://www.mdpi.com/2072-6643/10/3/381/s1, Table S1: Comparison of the concentrations of water-soluble forms of choline in milk samples in the Canadian trials, Table S2: Comparison of the concentrations of water-soluble forms of choline in milk samples in the Cambodian trial, and Table S3: Estimated dietary choline intake during pregnancy from a subset of Canadian participants.
