# Supplementary material for: Concentrations of Water-Soluble Forms of Choline in Human Milk from Lactating Women in Canada and Cambodia
Source: Nutrients. 2018 Mar 20;10(3):381. doi: 10.3390/nu10030381 (PMC5872799; doi:10.3390/nu10030381)
Supplement: Supplementary file 1 [file nutrients-10-00381-s001.pdf]

Supplementary Table S1. Comparison of the concentrations of water-soluble forms of choline in milk samples in the Canadian trials [1].

Comparison	*n*	Free choline	Phospho-choline	Glycerophospho-choline	Water-soluble choline [5]
Canadian trial 1, by treatment					
Placebo	79	153 ± 85	514 ± 193	394 ± 121	1061 ± 204
DHA, 400 mg/d	68	139 ± 57	525 ± 155	388 ± 131	1052 ± 211
P value [2]		0.533	0.702	0.652	0.772
Canadian trial 2, by treatment					
Vitamin D, 10 μg/d	54	143 ± 93	554 ± 241	439 ± 159	1136 ± 349
Vitamin D, 25 μg/d	48	152 ± 104	546 ± 216	415 ± 160	1122 ± 324
Vitamin D, 50 μg/d	52	164 ± 90	571 ± 238	425 ± 211	1158 ± 391
P value [3]		0.086	0.626	0.673	0.634
By Canadian trial					
Canadian trial 1	147	147 ± 73	519 ± 176	391 ± 125	1057 ± 207
Canadian trial 2	154	153 ± 103	551 ± 232	426 ± 178	1136 ± 369
P value [4]		0.604	0.169	0.078	0.140

[1] Data presented as mean ± SD (μmol/L), concentrations were quantified using liquid chromatography-tandem mass spectrometry; [2] Trial 1 compared docosahexaenoic acid (DHA) versus placebo and difference by treatment was assessed by independent samples Student's t test; [3] Trial 2 compared doses of vitamin D and difference by treatment was assessed by one-way ANOVA; [4] Trial 1 used hindmilk and trial 2 used a full breast expression, difference by trial was assessed by independent samples Student's t test; [5] Water-soluble choline corresponds to the sum of free choline, phosphocholine, and glycerophosphocholine.

Supplementary Table S2. Comparison of the concentrations of water-soluble forms of choline in milk samples in the Cambodian trial.

Comparison	*n*	Free choline	Phospho-choline	Glycerophospho-choline	Water-soluble choline [4]
By treatment [1]					
Placebo	22	135 ± 68	541 ± 194	393 ± 126	1069 ± 253
Thiamine, 2 g/L	26	150 ± 109	560 ± 186	387 ± 153	1098 ± 287
Thiamine, 8 g/L	19	146 ± 75	579 ± 244	389 ± 134	1115 ± 274
P value [2]		0.110	0.751	0.990	0.536
By weeks postpartum [3]					
r	67	0.094	-0.173	0.202	0.011
P value		0.447	0.161	0.101	0.930

[1] Data presented as mean ± SD (μmol/L), concentrations were quantified using liquid chromatography-tandem mass spectrometry; [2] The Cambodian trial compared doses of thiamine versus placebo and difference by treatment was assessed by one-way ANOVA; [3] Milk samples were collected between 3 – 28 weeks postpartum and the association with the concentrations of water-soluble forms of choline was determined using Pearson's correlation; [4] Water-soluble choline corresponds to the sum of free choline, phosphocholine, and glycerophosphocholine.

Supplementary Table S3. Estimated dietary choline intake during pregnancy from a subset of Canadian participants.

**Dietary intakes** [1] (*n* = 143)	**16 weeks of gestation**	**36 weeks of gestation**	**Difference** [5] *P* value	**Correlation** [6] r	*P* value
Free choline	92.2 ± 25.5	86.7 ± 23.5	0.175	0.544	< 0.001
Phosphocholine	24.5 ± 9.6	22.6 ± 10.7	0.156	0.479	< 0.001
Glycerophosphocholine	87.5 ± 35.3	92.3 ± 35.3	0.106	0.493	< 0.001
Water-soluble choline [2]	204.2 ± 61.5	202.6 ± 58.0	0.787	0.559	< 0.001
Phosphatidylcholine	185.2 ± 60.6	185.5 ± 66.0	0.958	0.505	< 0.001
Sphingomyelin	19.7 ± 6.3	20.4 ± 6.5	0.145	0.573	< 0.001
Lipid-soluble choline [3]	205.0 ± 65.3	205.9 ± 71.1	0.873	0.519	< 0.001
Total choline [4]	409.2 ± 102.2	408.5 ± 111.0	0.927	0.590	< 0.001

[1] Data presented as mean ± SD (mg/d), dietary choline intakes were estimated at 36 weeks of gestation using a food frequency questionnaire and the USDA database on choline content in common foods (version 2); [2] Water-soluble choline corresponds to the sum of free choline, phosphocholine, and glycerophosphocholine; [3] Lipid-soluble choline corresponds to the sum of phosphatidylcholine and sphingomyelin; [4] Total choline corresponds to the sum of all individual forms of choline; [5] Dependent samples Student's t test; [6] Pearson's correlation coefficients.
